# Altering Nitrogen Sources Affects Growth Carbon Costs in *Vachellia nilotica* Growing in Nutrient-Deficient Grassland Soils

**DOI:** 10.3390/plants10091762

**Published:** 2021-08-25

**Authors:** Nonkululeko Sithole, Zivanai Tsvuura, Kevin Kirkman, Anathi Magadlela

**Affiliations:** 1 School of Life Sciences, College of Agriculture, Engineering and Science, Westville Campus, University of KwaZulu-Natal, Private Bag X54001, Durban 4000, South Africa; lulekosithole@gmail.com; 2 School of Life Sciences, College of Agriculture, Engineering and Science, Pietermaritzburg Campus, University of KwaZulu-Natal, Private Bag X01, Scottsville 3209, South Africa; Tsvuuraz@ukzn.ac.za (Z.T.); KirkmanK@ukzn.ac.za (K.K.)

**Keywords:** KwaZulu-Natal grassland soils, P deficiency, *Vachellia* *nilotica*, N-fixing bacteria, *Mesorhizobium*

## Abstract

*Vachellianilotica* (L.) Willd. Ex Del. is a multipurpose leguminous tree that is common in grassland and savanna ecosystems in southern and eastern Africa. These ecosystem soils are reported to be acidic and nutrient-limited, specifically with regards to nitrogen (N) and phosphorus (P). The presence of this plant in these terrestrial ecosystems improves soil fertility benefiting the surrounding vegetation due to its ability to fix atmospheric N. This study seeks to understand the N-fixing bacteria symbiosis and physiological adaptations of *V. nilotica* in these acidic and nutrient-deficient KwaZulu-Natal soils. The soils used for this study were collected from the Ukulinga Grassland Nutrient Experiment located at the Ukulinga research farm of the University of KwaZulu-Natal, Pietermaritzburg, South Africa. Due to long-term soil nutrient addition treatments, these soils offered a diverse nutrient variation for better understanding the effects of acidity and nutrient variation on microbial symbiosis, plant nutrition, and biomass accumulation of *V. nilotica*. *V. nilotica* was able to maintain growth by relying on both atmospheric and soil-derived N across all treatments decreasing carbon (C) growth costs. There was an increased reliance on atmospheric-derived N of un-nodulated high N-treated plants. The plants grown in high N + P soils were able to nodulate with various species from the *Mesorhizobium* genus, which resulted in increased biomass compared to other plants. The results of this study show that *V. nilotica* can alter N sources to reduce C growth costs. In addition, both nodulating and free-living soil N_2_ fixing bacteria such as *Caulobacter rhizosphaerae*, *Sphingomonas* sp. and *Burkholderia contaminans* identified in the experimental soils may play an important role under P-deficient conditions.

## 1. Introduction

Grassland and savanna ecosystems in KwaZulu-Natal (KZN), South Africa, and elsewhere in the world are generally nutrient-poor, specifically with regards to the primary nutrients nitrogen (N) and phosphorus (P), which are essential for plant growth and development [1]. Dinitrogen (N_2_) accounts for about 78% of the atmosphere and is mostly unavailable for plant use [2]. Plants need N for the production of essential biomolecules such as nucleic acids, amino acids, and proteins [3]. The presence or incorporation of legume plants in natural and semi-natural ecosystems is important due to their ability to improve soil nutrient status in a cost-effective [4] and sustainable manner [5]. Legumes are characteristic in South African grassland and savanna ecosystems and form an important component of the N cycle [6]. Through the biological nitrogen fixation (BNF) process, legumes are able to fix N_2_ into plant usable forms such as NH_4_ [7]. The BNF process is mediated by species-specific rhizobia, a bacterium common in natural soils [8].

Legumes require large amounts of P for adenosine triphosphate (ATP) production, an energy unit needed during the BNF process [9]. Sixteen ATP molecules are reduced to adenosine diphosphate (ADP) when a single molecule of N_2_ is reduced to ammonia (NH_3_) [10]. Thus, where P is limiting, the nodules are highly reduced, which ultimately decreases the efficiency of BNF [11]. P is the most abundant and least mobile [12] essential nutrient in the soil and is often bound to cations such as iron (Fe) and aluminum (Al) oxides [13], making it unavailable for plant uptake and use [14]. Legumes have several adaptations enabling their survival in nutrient-deficient environments [15]. In addition to rhizobia- legume symbiosis, arbuscular mycorrhizal (AM) fungi form an additional symbiont resulting in a tripartite symbiosis [16]. The AM fungi enhance nutrient acquisition, including P [17].

Mutualistic associations are important in legume plant growth and development [18], and this was evident in a study conducted by [19] where the rhizobia and AM fungi improved the growth of *Vigna unguiculata* in P-deficient soils conditions through enhanced P uptake. The symbionts, in turn, receive a constant supply of photosynthates from the legume plant [20]. The expense of this symbiosis is costly to plants, especially where nutrients are limited [21]. This results in legumes altering their nitrogen source between atmospheric-derived N and soil-derived N to minimize the expense [22]. Various free-living and symbiotic bacteria have been reported to solubilize cation bound P making it available for plant use. These include strains from the *Bacillus*, *Azospirillum*, *Paenibacillus,* and *Azotobacter* genera [23,24,25]. *Vachellia nilotica*, previously known as *Acacia nilotica*, is a multipurpose leguminous plant with numerous ecological, social, and economic benefits across the world [26,27,28]. *V. nilotica* has a wide distribution in South African grassland and savannas ecosystems [27]. Research on microbe symbiosis and physiological adaptation of *V. nilotica* in nutrient-deficient soils of grassland and savanna ecosystems is limited. Consequently, this study seeks to investigate the N_2_-fixing bacteria, plant nutrition, carbon (C) costs and biomass accumulation in *V. nilotica* grown in KZN acidic soils with varying N and P nutrient status at the Ukulinga Grassland Nutrient Experiment (UGNE) located at the Ukulinga research farm of the University of KwaZulu-Natal, Pietermaritzburg, KZN province of South Africa. The proposed hypothesis was that *V. nilotica* will alter its N source preference to reduce growth C costs in P-deficient soils.

## 2. Results

### 2.1. Soil Characteristics

Percentage N concentration was significantly lower in the N1 soils compared to other soils (Supplementary Table S1). P concentration was significantly higher in the N1 + P, N2 + P, and N3 + P soils compared to other soils (Supplementary Table S1). However, the K concentration was significantly higher in the N1 and N3 soils (Supplementary Table S1). The exchangeable acidity was significantly higher in the N3 and N3 + P soils compared to the N1, N1 + P, and N2 + P treatments (Supplementary Table S1). In addition, the soils were acidic across all treatments with a pH below 5. The pH followed the same trend as the exchange acidity as the N3, and N3 + P soils were more acidic compared to soils N1, N1 + P, and N2 + P (Supplementary Table S1). The moisture factor was significantly lower in N1 compared to other soils (Supplementary Table S1).

### 2.2. Soil Bacteria and Plant Endophytic Bacterial Isolates Identification

The molecular identification of N-fixing and N-cycling bacteria in the experimental soils used as growth substrate included, *Caulobacter rhizosphaerae*, *Sphingomonas* sp. and *Burkholderia contaminans* with accession no. and similarity (%) detailed in Supplementary Table S2. *V. nilotica* grown in N2 + P and N3 + P soils were the only plants that were able to form a symbiotic association with the nitrogen-fixing bacteria. The 16S rRNA gene revealed that the symbionts were various strains from *Mesorhizobium* in both treatments (Supplementary Table S2).

### 2.3. Biomass and Mineral Nutrition

*V. nilotica* grown in N1 + P, N2 + P, and N3 + P soils had significantly higher total biomass compared to plants grown in N1, N2, and N3 soils (Table 1). The shoots had the highest biomass in N1 + P, N2 + P, and N3 + P soils compared to other soils (Table 1). A similar trend as the shoot biomass was observed in roots biomass as the roots had the highest biomass in N1 + P, N2 + P, and N3 + P soils. The leaves had the highest biomass in N1 + P, N2 + P, and N2 + P soils, followed by N1 soils (Table 1). The root biomass was higher in N2 + P and N3 + P soils compared to other soils (Table 1). The root: shoot ratio of *V. nilotica* grown in N3 soils was significantly higher and significantly lower in soils N1 and N2 soils (Table 1). P concentration was significantly higher in *V. nilotica* grown in N1 + P and N3 + P soils, followed by the *V. nilotica* grown in N2 + P soils. However, the N concentration was significantly higher in *V. nilotica* grown in N2 and N3 soils compared to other soils (Table 1).

### 2.4. Growth Kinetics

**Figure 1:**
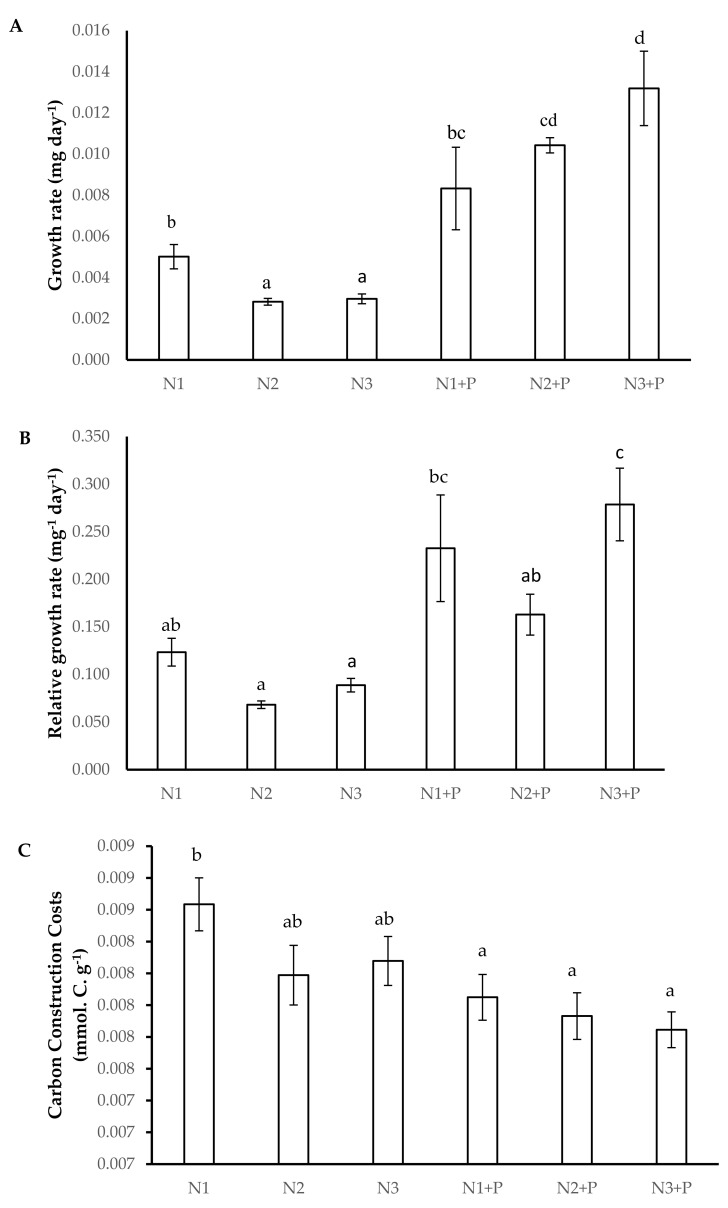
Growth kinetics ((**A**). Growth rate, (**B**). Relative growth rate, (**C**). Carbon construction costs) of 180-day-old *V. nilotica* saplings grown in Ukulinga Farm soils. Values represent the mean ± SE, based on *n* = 5. Significant differences (*p* < 0.05) among treatments are denoted by different superscript letters.

*V. nilotica* grown in N1 + P, N2 + P, and N3 + P soils had a significantly high growth rate compared to *V. nilotica* plants grown in other soils (Figure 1A). A significantly higher relative growth rate was observed in *V. nilotica* grown in N1 + P, N2 + P, and N3 + P soils (Figure 1B). The C-costs were significantly higher in N1 soils compared to other soils (Figure 1C).

### 2.5. N and P Nutrition

**Figure 2:**
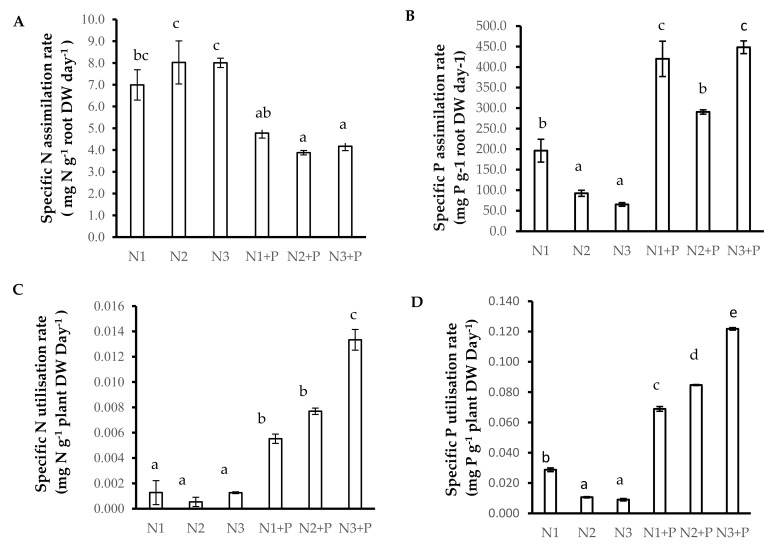
N and P use efficiency ((**A**). Specific N assimilation rate, (**B**). Specific P assimilation rate, (**C**). Specific N utilisation rate, (**D**). Specific P utilisation rate) of 180-day-old *V. nilotica* saplings grown in Ukulinga Farm soils. Values represent the mean ± SE, based on *n* = 5. Significant differences (*p* < 0.05) among treatments are denoted by different superscript letters.

Plants grown in N2 and N3 soils showed increased specific nitrogen absorption rate (SNAR), followed by plants grown in N1 and N1 + P soils, whereas the plants grown in N2 + P and N3 + P had the lowest SNAR (Figure 2A). The specific nitrogen utilization rate (SNUR) was significantly higher in N3 + P soils, followed by that of plants grown in N1 + P and N2 + P soils (Figure 2C). The specific phosphorus assimilation rate (SPAR) was significantly increased in N1 + P and N3 + P soils, followed by N1 and N2 + P soils and with N2 and N3 grown *V. nilotica* having a significantly lower SPAR (Figure 2B). The specific phosphorus utilization rate (SPUR) followed the same trend in low P concentration (N1, N2, and N3) soils, and a staggering significant increase in SPUR was observed in *V. nilotica* grown N1 + P, followed by N2 + P and the N3 + P (Figure 2D).

### 2.6. N Source Preference

**Figure 3:**
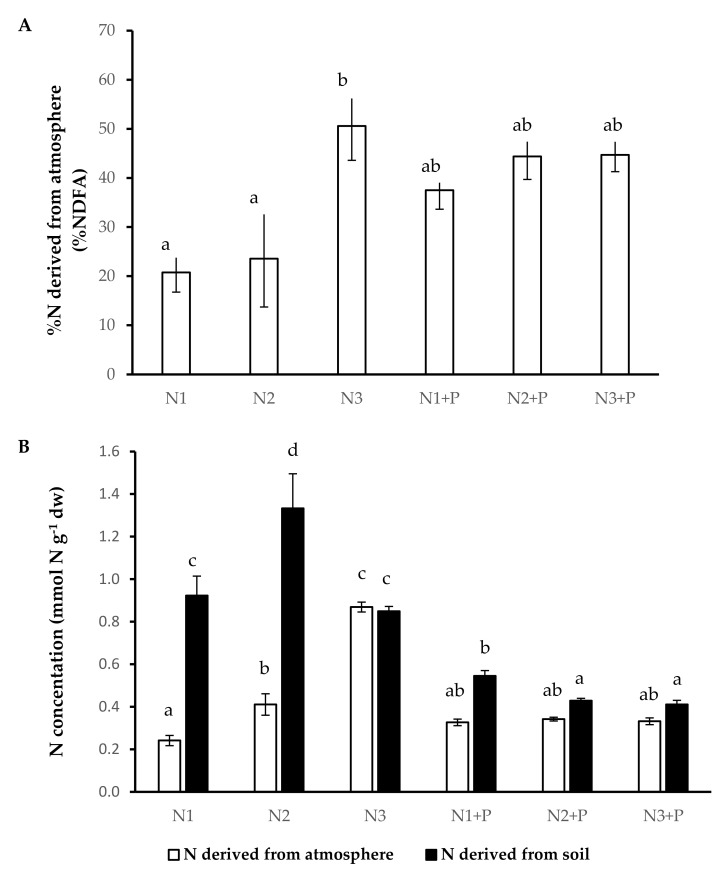
N source preferences ((**A**). Percentage N derived from the atmosphere, (**B**). Plant N concentration derived from atmosphere and Plant N concentration derived from soil) of 180-day-old *V. nilotica* saplings grown in Ukulinga Farm soils. Values represent the mean ± SE, based on *n* = 5. Significant differences (*p* < 0.05) among treatments are denoted by different superscript letters.

Plants grown in N3 soils obtained an almost equal amount of N from the soil (NDFS) as well as from the atmosphere (NDFA). The plants grown in N1 and N2 had significantly low rates of N from the atmosphere (%NDFA) with a significantly high dependence on NDFS compared to plants grown in other soils (Figure 3).

## 3. Discussion

Phosphorus limitation negatively affected nodulation of *V. nilotica* under low N (N1), intermediate N (N2), and high N (N3) treatments and in low N and high P (N1 + P) soils. Ferreira et al. [29] reported an increase in nodulation and biological N fixation in *Calopogonium mucunoides* Desv. grown in acidic soils (pH~4). This was different from our findings as *V. nilotica* failed to nodulate and relied more on nitrogen derived from the soil (NDFS) in acidic soils (N1, N2, N3, and N1 + P soils) with a pH between 4.1 and 4.8. This could be due to the reduced P levels in the soil, as P is important in regulating energy requirements for nodule formation and biological nitrogen fixation (BNF) [30]. Legumes can rely on actinomycetes and Gram-positive bacteria for nitrogen fixation without nodulation [31,32]. This was evident in a study conducted by [33], who isolated various species belonging to the *Streptomyces* genus from rhizospheric soils of kidney bean, chickpea, soybean, pea, and lentil, which fixed atmospheric N resulting in increased N available for legume uptake and use. This concurs with our findings as the increased level of N (approximately 50%) derived from the atmosphere (NDFA) was observed in *V. nilotica* grown in N3 soils. These findings suggest that soil free-living actinomycetes (*Caulobacter rhizosphaerae*, *Sphingomonas* sp. and *Burkholderia contaminans*) might have contributed the NDFA as the plants did not develop any nodules.

In high P concentration soils with intermediate and high N levels, the 16S rRNA gene revealed that *V. nilotica* was nodulated with highly effective symbionts consisting of various strains of *Mesorhizobium* spp. However, the NDFA ranged between 40% and 50%. The plants in these soils also relied on N derived from the soil (NDFS) as it is cost-effective to assimilate inorganic N than to fix N_2_ from the atmosphere [20]. This is also supported by the significantly low C costs in *V. nilotica* grown in the P-rich soils.

P deficiency decreases the above-ground biomass [34] and invests in below-ground biomass when nutrients are scarce in order to maximize the surface area for nutrient acquisition through altered root architecture [35]. This concurs with our findings as a significant reduction in shoot biomass was observed in *V. nilotica* grown in P-deficient soils (N2 and N3) while significantly increasing their root biomass resulting in increased specific N assimilation and utilization rate. In addition, N1 grown plants had increased SPAR and SPUR and showed a relatively high total biomass when compared to the plants grown in N2 and N3. In addition to the increased root biomass, these plants may have established symbiosis with AM fungi as the N1 soils had significantly low P concentration. AM fungi can enhance nutrient uptake under nutrient-deficient conditions [36]. However, this was not analyzed in the current study. It is also important to note that the low N concentration levels in N1 soils could have facilitated the survival of AM fungi. Fungal diversity and abundance tend to decline in high N concentration environments [37].

In addition, P plays a vital role in adenosine triphosphate (ATP) production, which is essential for biological nitrogen fixation (BNF). This could explain the inability of *V. nilotica* to develop nodules in low, moderate, and high N (N1, N2, and N3) soils. Inversely, the total biomass of *V. nilotica* grown in P enriched soils was higher due to the increased level of SPAR and SPUR. N1, N2, and N3 soils were highly acidic. In highly acidic soils, soil P precipitates as orthophosphate and is adsorbed by Fe and Al oxides making the P unavailable for plant assimilation and utilization [24]. N1 and N2 soils had significantly low P concentrations compared to N3 soils. However, *V. nilotica* grown in N1 and N2 soils had significantly high P content compared to *V. nilotica* grown in N3 soils. These findings may be due to increased levels of N in the N3 soil as [38] reported a decrease in P solubilizing bacteria due to increased N levels.

## 4. Materials and Methods

### 4.1. Study Site

Soil samples were collected from the UGNE located at the Ukulinga research farm of the University of KwaZulu-Natal in Pietermaritzburg, South Africa (29°24′ E, 30°24′ S). The altitudinal gradient in which the experiment is set up ranges from 838 to 847 m above sea level [39]. The mean annual precipitation and temperature of the area are approximately 838 mm and 18 °C, respectively [40].

### 4.2. Experimental Design

The long-term veld fertilized trials (VFT) were initiated in 1951 through the manipulation of nitrogen (N), phosphorus (P), and lime (L). There were initially 96 plots from the years 1951–2019, and each plot was 9.0 × 2.7 m^2^ in size with a 1 m spacing between plots. The experiment was replicated in three blocks, each block containing 32 plots, resulting in a 4 × 2^3^ factorial design. From the 3 blocks, the plots fertilized with N in the form of limestone ammonium nitrate (LAN) and P in the form of superphosphate were used in this study. Three levels of 28% N (N1 = 210 kg/ha/season; N2 = 421 kg/ha/season and N3 = 632 kg/ha/season) fertilizer was applied two times a year. These three N levels were also applied in combination with one level of 11.3% P (336 kg/ha/season) (N1 + P, N2 + P, and N3 + P), which was applied once a year, adding up to six treatments used for this study.

### 4.3. Soil Characteristics Analysis and Bacterial Identification

For each treatment, five soil samples were collected within the three blocks at a depth of ~0–30 cm to avoid any damage to the ongoing fertilization trials. Five subsamples of 50 g of soil from each treatment were collected and sent for P, N, K, pH, acidity exchange, and total cation analysis at the Analytical Services Unit of the KwaZulu-Natal Department of Agriculture and Rural Development at Cedara, South Africa. Soil moisture factor (g/g) was calculated based on the ratio of air-dried:oven-dried soils to calculate values relative to oven-dry mass at 105 °C as detailed by [41]. An additional five soil samples (250–300 g) from each treatment were used for microbial identification, where the bacterial DNA was extracted using a modified boiling procedure described by [42]. The bacterial DNA amplification using the 16S rRNA gene, sequencing, and identification was performed as detailed in [43]. The bulk of the remaining soils from each treatment were pooled for uniformity and used for the seedling growth experiment as a growth substrate.

### 4.4. Seed Germination and Growth Conditions

*V. nilotica* seeds were collected from Mposhini Nature Reserve near Pietermaritzburg, South Africa. The experiment was conducted under ambient conditions in a greenhouse at the University of KwaZulu-Natal botanical gardens in Pietermaritzburg. The greenhouse conditions were 12 to 14 °C and 30 to 35 °C night and daytime temperatures, respectively. Humidity ranged from 70% to 80%, and irradiance was 35% of full sunlight (i.e., 415.6 µmol m^2^ s^1^).

Prior to germination, the seeds were soaked in 15% sodium hypochlorite for 20 min. Thereafter, seeds were rinsed five times with distilled water and then placed in Petri dishes layered with filter paper for germination. The seeds were watered every day until seedling emergence. Thereafter, seedlings were planted at a depth of 1–2 cm in 15 cm diameter plastic pots containing soil from the VFT. The experiment was a random block design with the six soil nutrient treatments. Each treatment had 20 replicates. Plants were watered every two days in the afternoon, depending on the climate conditions.

### 4.5. Plant Harvesting and Nutrient Analysis

The initial harvest for determination of initial plant size prior to induction of treatment effects was undertaken 30 days after seedling emergence, while final harvests occurred 180 days after seedling emergence. During each harvest, 10 plants were rinsed with distilled water and separated into leaves, stems, roots, and nodules and thereafter oven-dried at 65 °C for 4 days. Their dry weights were recorded; thereafter, plants were ground to powder. The ground plant material was put in 2 mL Eppendorf tubes and sent for C and N isotope analysis and P analysis at the Archaeometry Department at the University of Cape Town and at the Central Analytical Facilities of Stellenbosch University, respectively, both in South Africa.

From the remaining plant, root nodules were harvested for bacterial extraction. Root nodules were rinsed with distilled water, then sterilized with ethanol (70% (*v*/*v*)) for 30 s and with sodium hypochlorite solution (3.5% (*v*/*v*) for 3 min. Thereafter, nodules were rinsed 10× with distilled water and stored in airtight vials containing silica gel and cotton wool. The vials were then stored at −4 °C before bacterial extraction, culturing, and sequencing.

### 4.6. Bacterial Extraction and Identification

Prior to bacterial extraction, the vials containing nodules were transferred into 2 mL Eppendorf tubes containing distilled water and left overnight to absorb water at −4 °C. The nodules were again sterilized (70% ethanol for 30 s; 3.5% sodium hypochlorite solution for 3 min) and thereafter rinsed 10× with distilled water. The nodule samples were then crushed in 15% glycerol solution. The turbid nodule solution in 15% glycerol was then streaked in plates containing yeast mannitol agar (YMA) containing 0.5 g/L yeast extract (Biolab), 10 g/L mannitol (Saarchem), 0.5 g/L di-potassium hydrogen orthophosphate (K_2_HPO_4_, Biolab), 0.2 g/L magnesium sulfate heptahydrate (MgSO_4_.7H_2_O, Biolab), 0.1 g/L sodium chloride (NaCl, Biolab), 15 g/L bacteriological agar (Biolab) and incubated at 28 °C. The bacteria were re-streaked into fresh plates until pure colonies were obtained.

The pure bacterial colonies were amplified using a portion of 16-S rRNA gene, 27F (5′-AGAGTTTGATCCTGGCTCAG-3′) and 1492R (5′-GGTTACCTTGTTACGACTT-3′). The PCR experiment volumes were 50 µL reaction containing sterile milliQ water, primers (10 µM), DNTPs (2 mM), SuperTherm Taq DNA polymerase (50–100 ng), MgCl_2_ (25 mM), 1 µL of pure bacterial colony, and BSA (10 mg/mL). The PCR cycle conditions consisted of initial denaturation at 95 °C for 5 min, 30 cycles of denaturation at 95 °C for 1 min, annealing at 55 °C for 1 min, extension at 72 °C for 1 min, and a final elongation step for 72 °C for 10 min. The results were viewed in 1% (*m*/*v*) agarose gel electrophoresis using TAE buffer and run at 100 V for 20 min. Thereafter, amplified products were sent for sequencing at the Central Analytical Facilities at Stellenbosch University. The resulting sequences were edited and subjected to BLASTN searches for identification (National Center for Biotechnology Information, NCBI, https://www.ncbi.nlm.nih.gov/genbank/, accessed on 16 June 2020).

### 4.7. Growth Calculations

#### 4.7.1. Calculation of N Derived from the Atmosphere

The isotopic ratio of N was calculated as δ = 1000 (*R*_sample_/*R*_standard_), where *R* is the molar ratio of the heavier to the lighter isotope of the samples and standards. Between 2.10 and 2.20 mg of each milled sample were weighed into 8 × 5 mm tin capsules (Elemental Micro-analysis, Devon, UK) on a Sartorius microbalance (Goettingen, Germany). The samples were then combusted in a Fisons NA 1500 (Series 2) CHN analyzer (Fisons Instruments SpA, Milan, Italy). The nitrogen isotope values for the N gas released were determined on a Finnigan Matt 252 mass spectrometer (Finnigan MAT GmbH, Bremen, Germany), which was connected to a CHN analyzer by a Finnigan MAT Conflo control unit. Five standards were used to correct the samples for machine drift, namely, two in-house standards (Merck Gel and Nasturtium) and the IAEA (International Atomic Energy Agency) standard (NH_4_)2SO_4_. Percent N derived from the atmosphere was calculated as:

%NDFA = 100 ((*δ*^15^*N* reference plant − *δ*^15^*N* legume)/(*δ*^15^*N* reference plant − *β*))

where NDFA is the N derived from the atmosphere. The *β* value represents the *δ*^15^N natural abundance of the N derived from biological N_2_ fixation. *V. nilotica* grown in N-free culture was determined to be −2.58‰.

#### 4.7.2. Calculation of the Specific N/P Absorption Rate

Specific nitrogen absorption rate (SNAR) values were obtained by calculating the total N absorbed by the plant through the roots (mg Ng^−1^ root dw day^−1^):

SNAR = (N_2_ − N_1_)/(t_2_ − t_1_) ∗ [(log_e_ R_2_ − log_e_ R_1_)/(R_2_ − R_1_)]

SPAR = (P_2_ − P_1_)/(t_2_ − t_1_) ∗ [(log_e_ R_2_ − log_e_ R_1_)/(R_2_ − R_1_)]

where N and P denote the total nitrogen and phosphorus content in the plant, t is the time it took for the plant to grow, and R, the root dry weight, is as described in [44].

#### 4.7.3. Calculation of the Specific N/P Utilization Rate

Specific nitrogen utilization rate (SNUR) values were obtained by calculating the dry weight acquired by the plant during nitrogen uptake (g dw mg^−1^ N day^−1^):

SNUR = (W_2_ − W_1_)/(t_2_ − t_1_) ∗ [(log_e_ N_2_ − log_e_ N_1_)/(N_2_ − N_1_)]

SPUR = (W_2_ − W_1_)/(t_2_ − t_1_) ∗ [(log_e_ P_2_ − log_e_ P_1_)/(P_2_ − P_1_)]

where W is the plant dry weight [44], and the other parameters are as defined in the SNAR and SPAR equations.

#### 4.7.4. Relative Growth Rate

Relative growth rate (RGR) was calculated according to [45]

RGR = [(ln W_2_ − ln W_1_)/(t_2_ − t_1_)]

where W denotes the dry weights and t, the time it took for the plant to grow, i.e., from day 30 to day 180.

#### 4.7.5. Carbon Construction Costs

Carbon construction costs (C_w_) were obtained from the formula used by [46], which was derived from [47] as follows:

C_w_ = (C + *k*N/14 ∗ 180/24) (1/0.89) (6000/180)

C_w_ denotes the total carbon construction cost (mmol C g^−1^ dry weight (DW)) of the tissues, C is the total concentration of carbon (mmol C g^−1^), k is the reduction state of the N substrate (for NH_3_ = −3), and N is the total organic nitrogen content of the tissue (g DW^−1^) as described by [48] The numerical value 14 is the atomic mass of nitrogen, 180 is a conversion factor from moles to grams of glucose. The amount of electrons in a glucose molecule that are available are 24, while 0.89 is an estimate of growth efficiency [48], and the fraction 6000/180 is a constant conversion factor from g^−1^ dry weight to mmol C g^−1^ DW for glucose.

#### 4.7.6. Statistical Analysis

IBM SPSS Statistics v. 24 was used to analyze the effects of N and P concentration variability in the nutrient trials on *V. nilotica* biomass, soil nutrient and fungal status, plant mineral nutrition, and growth kinetics using one-way analysis of variance (ANOVA). Where the assumptions of normality were not met, a Kruskal–Wallis test was performed, and where the variances were significantly different, a Bonferroni’s post hoc test was performed to separate the means (≤0.05)**.**

## 5. Conclusions

*V. nilotica* invested in below-ground biomass during nutrient deficiency to maximize the surface area for nutrient acquisition through altered root architecture. This legume plant was able to maintain its growth by relying on both atmospheric and soil-derived N across all treatments. The increased reliance of un-nodulated *V. nilotica* on atmospheric-derived N highlights the significance of free-living N_2_ fixing and cycling bacteria (*Caulobacter rhizosphaerae*, *Sphingomonas* sp. and *Burkholderia contaminans*) under P-deficient conditions. In addition, *Mesorhizobium* spp. may be able to withstand soil acidity in savanna soils.

## Figures and Tables

**Table 1:** Biomass and mineral nutrition of 180-day-old *V. nilotica* saplings grown in Ukulinga Farm soils receiving N and P fertilizer treatments Values represent mean ± SE, based on *n* = 5. Significant differences (*p* < 0.05) among treatments are denoted by different superscript letters.

	Treatment Trials					
Parameter	N1	N2	N3	N1 + P	N2 + P	N3 + P
Biomass (g)						
Total plant	0.90 ± 0.11 ^ab^	0.51 ± 0.03 ^a^	0.53 ± 0.04 ^a^	1.50 ± 0.36 ^ac^	1.88 ± 0.25 ^bc^	2.38 ± 0.33 ^c^
Leaves	0.36 ± 0.05 ^b^	0.17 ± 0.01 ^a^	0.13 ± 0.02 ^a^	0.49 ± 0.15 ^bd^	0.70 ± 0.10 ^cd^	0.87 ± 0.09 ^c^
Shoot	0.28 ± 0.04 ^ab^	0.14 ± 02 ^a^	0.19 ± 0.02 ^a^	0.50 ± 0.10 ^ac^	0.58 ± 0.10 ^bc^	0.74 ± 0.18 ^c^
Roots	0.27 ± 0.04 ^ab^	0.20 ± 0.02 ^a^	0.21 ± 0.01 ^ab^	0.51 ± 0.13 ^bc^	0.60 ± 0.05 ^c^	0.76 ± 0.10 ^c^
Root: shoot ratio	0.43 ± 0.05 ^a^	0.66 ± 0.05 ^a^	0.69 ± 0.06 ^b^	0.53 ± 0.09 ^ab^	0.49 ± 0.03 ^ab^	0.49 ± 0.05 ^ab^
Mineral nutrition						
Total plant N (mmol N g^−1^)	1.16 ± 0.12 ^a^	1.74 ± 0.21 ^b^	1.72 ± 0.05 ^b^	0.87 ± 0.04 ^a^	0.77 ± 0.02 ^a^	0.74 ± 0.04 ^a^
Standard corrected ^15^N/^14^N	2.88 ± 0.27 ^c^	2.69 ± 0.62 ^ac^	0.83 ± 0.23 ^ab^	1.73 ± 0.37 ^ac^	1.25 ± 0.34 ^ab^	1.23 ± 0.17 ^ab^
Total plant P (µmol P g^−1^)	32.52 ± 4.60 ^b^	19.98 ± 1.62 ^ab^	13.92 ± 0.93 ^a^	75.76 ± 7.76 ^d^	57.08 ± 1.03 ^c^	79.16 ± 2.74 ^d^

## Supplementary Materials

The following are available online at https://www.mdpi.com/article/10.3390/plants10091762/s1, Table S1: title: Soil N, P, and K concentrations, exchange acidity, moisture factor, and pH determined from the nutrient addition trials at Ukulinga Farm, KwaZulu-Natal. Values represent mean ± SE, based on n = 4. Significant differences (*p* < 0.05) among treatments are denoted by different superscript letters. Table S2: title: The molecular identification of soil and plant isolated nitrogen-fixing bacteria.
