# Supplementary material for: Altering Nitrogen Sources Affects Growth Carbon Costs in Vachellia nilotica Growing in Nutrient-Deficient Grassland Soils
Source: Plants (Basel). 2021 Aug 25;10(9):1762. doi: 10.3390/plants10091762 (PMC8470937; doi:10.3390/plants10091762)
Supplement: Supplementary file 1 [file plants-10-01762-s001.zip › plants-1331978-supplementary.pdf]

Supplementary Table S1. Soil characteristics determined from the nutrient addition trials at Ukulinga Farm, KwaZulu-Natal. Values represent mean ± SE, based on n = 4. Significant differences ($p < 0.05$) among treatments are denoted by different superscript letters.

	**Treatment trials**					
**Parameter**	**N1**	**N2**	**N3**	**N1+P**	**N2+P**	**N3+P**
**Soil macronutrients (µmol g^{-1})**						
Leco N concentration	$0.27±0.01^{a}$	$0.32±0.01^{c}$	$0.31±0.00^{bc}$	$0.31±0.01^{bc}$	$0.28±0.01^{ab}$	$0.29±0.01^{ac}$
P concentration	$0.07±0.013^{a}$	$0.16±0.05^{bc}$	$0.10±0.01^{ab}$	$0.21±0.01^{c}$	$0.20±0.04^{c}$	$0.22±0.04^{c}$
K concentration	$3.81±0.58^{b}$	$3.07±0.58^{ab}$	$4.29±0.81^{b}$	$3.14±0.27^{ab}$	$1.74±1.45^{a}$	$1.45±0.14^{a}$
**Relative acidity**						
Exchangeable acidity (cmmol L^{-1})	$0.22±0.06^{a}$	$0.75±0.30^{b}$	$1.57±0.23^{c}$	$0.13±0.01^{a}$	$0.23±0.05^{a}$	$1.02±0.23^{bc}$
Moisture factor (g/g)	$1.07±0.03^{a}$	$1.08±0.00^{b}$	$1.08±0.00^{b}$	$1.08±0.00^{b}$	$1.08±0.00^{ab}$	$1.08±0.00^{ab}$
pH (KCl)	$4.67±0.07^{bc}$	$4.46±0.09^{ab}$	$4.12±0.04^{a}$	$4.77±0.04^{bc}$	$5.01±0.37^{c}$	$4.18±0.04^{ab}$
pH (H_20)	$5.63±0.07^{c}$	$5.45±0.10^{bc}$	$4.61±0.14^{a}$	$5.72±0.06^{c}$	$5.86±0.27^{c}$	$4.91±0.12^{ab}$

Supplementary Table S2. The molecular identification of soil and plant isolated nitrogen-fixing bacteria.

**Nitrogen-fixing bacteria**			Treatment (s)
**Strains**	**Accession No.**	**Similarity (%)**	

**Soil isolated nitrogen-fixing bacteria**			
Caulobacter rhizosphaerae strain IMCC34905	MK 138628	97.38	N1, N2, N3, N1 + P, N2 + P, and N3 + P
Sphingomonas sp. N-9	LC 101917	97.82	N1, N2, N3
Burkholderia contaminans strain J8A6SARS	MT 409575	99.01	N1 + P, N2 +P, and N3 + P
**Plant root nodule isolated nitrogen-fixing bacteria**			
Mesorhizobium sp. BwlS3– 11	KX 959579	98.01	N2 + P
Mesorhizobium sp. strain BwlS3-17	KX 959577	98.00	N3 + P
